# Survival Modeling on the Determinants of Time to Recovery from Obstetric Fistula: The Case of Mekelle Hamlin Fistula Center, Ethiopia

**DOI:** 10.1155/2022/8313575

**Published:** 2022-11-14

**Authors:** Abera Molla Bihon, Henok Kumsa Meikena, Selamawit Serka

**Affiliations:** ^1^Department of Statistics, College of Natural and Computational Sciences, Hawassa University, Ethiopia; ^2^Department of Midwifery, College of Health Sciences, Woldia University, Ethiopia

## Abstract

**Background:**

An obstetric fistula is an abnormal opening between the vagina, rectum, and/or bladder. Obstetric fistula has a devastating impact on women's physical, social, and psychological health. Despite the numerous health consequences in developing countries, including Ethiopia, there have been few studies on the determinants of time to recovery from obstetric fistula. Therefore, this study is aimed at addressing the gap.

**Methods:**

A retrospective cohort study was employed to include 328 randomly selected records of women admitted for obstetric fistula treatment at Mekelle Hamlin Fistula Center from January 2015 to 2020. Data collected from the medical records was coded and entered into SPSS software version 20 and exported to STATA 10 and R statistical software for data cleaning and data analysis. The Kaplan-Meier and log-rank tests were computed to explore the data. The log-logistic inverse Gaussian shared frailty model was employed using a 95% CI, and variables with a *p* value < 0.05 were declared as determinants of recovery time.

**Results:**

Of 328 fistula patients, 293 (89.33%) were physically cured. The Kaplan-Meier result showed that the overall mean and median survival time of time to recovery from obstetric fistula patients at Mekelle Hamlin Fistula Center is 42 and 33 days, respectively. In a log-logistic inverse Gaussian shared frailty model analysis, extensive fistula size (AHR : 1.282; 95% CI = 1.175-1.388), secondary and above education level (AHR : 0.830; 95% CI = 0.693-0.967), rural residence (AHR : 1.357; 95% CI = 1.236-1.479), and physiotherapy use (AHR : 0.801, 95% CI = 0662-0.940, 95% CI = 1.175-1.388) were statistically significant predictors of recovery from obstetrics fistula.

**Conclusion:**

Rural place of residence, home delivery, and large and extensive size of the fistula prolong the timing of healing from the obstetric fistula. However, having tall height, physiotherapy treatment, secondary and above-educated women, and RVF type of fistula has a short time of healing for obstetric fistula in Mekelle Hamlin Fistula Center. Therefore, we recommend that health professionals promote institutional delivery and physiotherapy, shorten the duration of catheterization, and manage urine incontinence. In addition, we recommend that the regional health bureau promotes female education and pregnancy after 18 years. The survival probability of patients with obstetric fistulas is better predicted by the log-logistic inverse Gaussian shared frailty model. Therefore, it would be good for future researchers to take this model into account.

## 1. Background

Obstetric fistula is a medical condition in which a hole develops in the birth canal as a result of childbirth. This can be between the vagina and rectum, ureter, or bladder [1]. Obstetric fistula is one of the most devastating medical disabilities afflicting women due to lack of intervention for prolonged or obstructed labor [2, 3]. In 2006, the WHO estimated that more than 2 million women throughout the globe live with untreated fistulas and that between 50,000 and 100,000 new women develop obstetric fistulas each year [3, 4]. Half of these cases of obstetric fistula are located in sub-Saharan Africa and South Asia. The prevalence of obstetric fistula in sub-Saharan Africa and South Asia was 1.6 and 1.2 per 1000 women of reproductive age, respectively [5].

Ethiopia is one of the developing countries with poor maternal health care that leads over 100,000 girls to live with a fistula, and another 9,000 cases develop annually [6]. Moreover, Ethiopian demographic health survey showed a lifetime prevalence rate of obstetric fistula of 10.6 per 1000 cases among women who had ever given birth [7]. Similarly, the Tigray Region was the area with the highest prevalence rate of obstetric fistulae (11 per 1000 women) [8].

Obstetric fistula is associated with devastating physical and medical consequences like paralysis of lower limbs and cessation of menstruation, vaginal scarring, failure to conceive, stone formation, urine incontinence, kidney disease, and renal failure. Furthermore, it is linked with social and psychological problems like feelings of shame, social isolation and segregation, divorce, depression, and lack of social support [9–11].

The recovery time of obstetric fistula patients depends on different factors such as height, weight, age, size of fistula, educational status, duration of labor, place of delivery, use of antibiotics, time of seeking care, and residence [12–16]. Despite the high public health impact and variation in determinants of fistula recovery in Ethiopia, specifically in the Mekelle Hamlin Fistula Center, there have been few studies on survival modeling on the determinants of time to recovery from obstetric fistula. Thus, this study is aimed at addressing the gap.

## 2. Methods

### 2.1. Study Design, Area, and Population

An institutional-based retrospective cohort study was conducted at Mekelle Hamlin Fistula Center. Mekelle Hamlin Fistula Center was established in February 2006 and is located in Mekelle City, Tigray Region, Ethiopia. It is one of the five mini fistula hospitals constructed to provide preventive, curative, and rehabilitative services to clients coming from urban and rural areas of Tigray and the surrounding regions (Northern Amara and Northwest Afar). This institution has been working with the regional health bureau as well as local and international nongovernmental organizations to reduce maternal morbidity and mortality secondary to fistula. According to the 2020 Mekelle Hamlin Fistula Center's report, 2908 patients were admitted for treatment of fistula. In addition, 155 patients were referred to the Addis Ababa Fistula Center in the period between 2006 and 2020 [17]. This study used medical records of obstetric fistula women from January 2015 to January 2020.

### 2.2. Eligibility Criteria

Obstetric fistula patients who were diagnosed, admitted to the center, and operated for obstetric fistula at Mekelle Hamlin Fistula Center from January 2015 to January 2020 and patients who had recovered or censored during this interval were included in the study. However, incomplete medical records of the baseline and follow-up data at least for the main exposure and outcome variables, all cases of fistula with a history of previous repair other than MHFC, women other than obstetric fistula, and women who came with a successfully closed fistula at the time of diagnosis were excluded from this study.

### 2.3. Sample Size Determination and Sampling Technique

Sample size was calculated using Cochran's formula [18]. Where *n* is the required sample size, *N* (1120) is the total population size from January 2015 to January 2020, and *Z*_*α*/2_ is the critical value of standard normal distributed variable at significance level *α* = 5%, let the maximum allowable difference between the maximum likelihood estimate and the unknown population parameter denoted by *d* = 0.0309. And *p* = 86.7% was from the pilot study result proportion of recovered (cured) fistula patients at MHFC. And *q* = (1 − *p*) = 13.7 was an estimated proportion of not recovered (censored) patients in the center. Finally, the required sample was 328 patients. One thousand one hundred twenty fistula patients were treated in the hospital, from January 2015 to March 2020; of these, 328 obstetric patients were selected randomly using the computer method.

### 2.4. Study Variable

#### 2.4.1. Dependent Variable

It is continuous and describes the length of hospital stay time in days. The response variable for the *i*^th^ individual is represented by Yi, and it measures duration to event and is defined by status variable (event or censoring variable). Survival time measures the follow-up of time from a defined starting point to the occurrence of a given event. This observation time has two components: the beginning point of the study time and the observation of time to the end. In survival analysis, the outcome of interest (recovery) is the duration of time until physically cured measured in days. Time to recovery is defined as time from surgery to recovery. Patients who did not recover during the study period, lost at follow up or death were considered as censored observations.

#### 2.4.2. Independent Variables

Covariates could affect the time to recovery from obstetric fistula classified as sociodemographic variables such as age at delivery, height, weight, BMI, place of residence, marital status, educational status, and economic dependence, whereas antibiotic use, physiotherapy, outcome of delivery, incontinence of urine, duration of catheterization, duration of labor, parity, place of delivery, fistula type and size, mode of delivery, and surgery approach were considered as obstetric variables.

### 2.5. Operational Definitions

- Time to recovery: the number of days from the time starting of the surgery performed for obstetric fistula patient in the hospital until to the patient discharged from the hospital
- Recovery: the condition of being healthy after somebody become ill or physically damage. In short, it is the condition of physically cure after patient taken surgery

### 2.6. Data Analysis

Data collected from the medical records was coded and entered into SPSS software version 20 and exported to STATA 10 and R statistical software for data cleaning and data analysis. Survival analysis was used to estimate the average (median) recovery time and to identify determinants of recovery time from obstetric fistula. The Kaplan-Meier test and the log-rank test were used to estimate the distribution of recovery time and to observe the experience of recovery time among different levels of categorical variables, respectively. The Cox proportional hazards model is a semiparametric model for fitting survival data, which describes the relationship between the event incidence, as expressed by the hazard function, and covariates that influence survival time. In order to use the Cox model, it has to be checked whether the assumption of whether the effects of covariates on hazard ratio remain constant over time. This is a vital assumption of the proportional hazards model and must be assessed for each covariate. Furthermore, Schoenfeld's residuals are employed to assess the assumption.

In this study, parametric survival models were also employed. Weibull AFT, log-normal AFT, and log-logistic AFT models were fitted using these data. And it used the AIC criteria to compare various candidates for parametric AFT and frailty models. Finally, the model with the smallest AIC value is considered a better fit. The procedure of model building was as follows. First, display the AIC value of each distribution on both the AFT and shared frailty models. Secondly, select the model that has the smallest AIC value. Thirdly, univariate analysis for the selected model in the first step and variables with a *p* value < 0.25 were candidates for multivariate analysis of the final model. Lastly, the selected survival model using a 95% CI and variables with a *p* value < 0.05 were declared as determinants of the recovery time of obstetric fistula. Additionally, the graphical methods are also used to check if a parametric distribution fits the observed data. A quantile-quantile plot was made to check if the accelerated failure time model provides adequate fitness to the data. The Cox-Snell residual plot was also used to check the overall fitness of the model (supplementary file).

## 3. Result

### 3.1. Sociodemographic Characteristics of Obstetric Fistula Patients

Three hundred twenty-eight medical cards of obstetric fistula patient were reviewed, 35 (10.67%) were censored at the end of the follow-up, and 293 (89.33%) of women were physically cured. Moreover, we look that the women age of delivery below 18 years old is 14.02% (46). Regarding the place of residence, majority of the obstetric fistula patients were 262 (79.87%) rural dwellers. One hundred fifty-nine (48.47%) of the obstetric fistula women have no formal education. The majority of obstetric fistula women had a large fistula hole (Table 1).

### 3.2. Obstetric and Fistula Characteristics of Obstetric Fistula Patients

Of the total obstetric fistula patients, the majority of obstetric fistula patients were multiparous (268, 81.7%). Sixty-nine (24.2%) obstetric fistula patients' outcomes of labor were stillbirth. Moreover, the majority of obstetric fistula patients gave birth vaginally (242, 73.8%) (Table 2).

### 3.3. Nonparametric Survival Analysis

#### 3.3.1. Kaplan-Meier Estimate of Time to Recovery from Obstetric Fistula

Nonparametric survival analysis was employed to visualize the survival of time to recovery of obstetric fistula patient from obstetric fistula under different levels of the factors. Moreover, survival time distributions for time to recovery are estimated for each group using the K-M method. In order to compare the survival curves of two or more groups, the log-rank test and generalized Wilcoxon test have been employed. Furthermore, the minimum and maximum recovery time of obstetric patients at Mekelle Hamlin Fistula Center was 4 days and 219 days, respectively, and available on supplementary file Table 3. The highest median survival time of time to recovery for obstetric fistula women age group was less than 18 years, which was 90 days, whereas the obstetric fistula women age group of above 30 years was the smallest median survival time (21 days) of time to recovery (Table 3).

**Figure 1:**
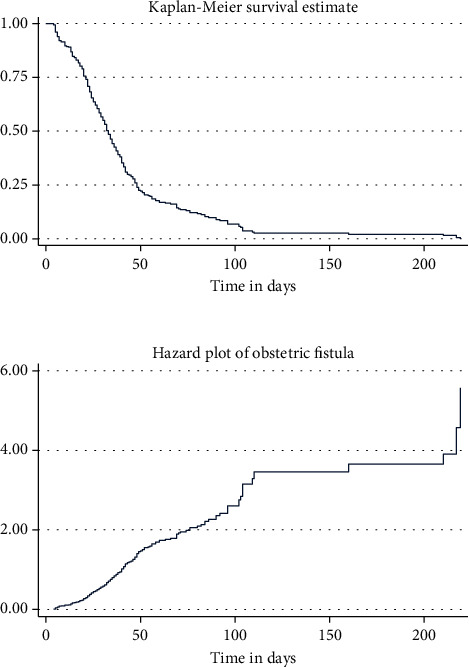
The K-M plots of the survival and hazard functions of recovery from obstetric fistula patients at MHFC (January 2015–January 2020).

The plots of the K-M curve to the survival and hazard experience of time to recovery from obstetric fistula are shown in Figure 1. The survival plot decreases at an increasing rate at the beginning and decreases at a decreasing rate later. This implies that most of the obstetric fistula patients were physically cured in a short period of time after starting intervention in the center. On the other hand, small numbers of obstetric fistula patients were physically cured after a long period of time. In addition, the K-M estimator survival curve can be used to estimate survivor function among different strata or groups of covariates. Separate graphs of the estimates of the K-M survivor functions for different categorical variables are available in the supplementary file (figure 2–21).

#### 3.3.2. Comparison of Survival Experiences of Obstetric Fistula Patients

Based on Tables 3 and 4, the log-rank test for survival difference was highly significant. The log-rank test shows that there was a significant difference in survival experience among groups for the height of patients, weight, age at delivery, marital status, educational status, parity, residence, economic dependence, antibiotic use, BMI, physiotherapy use, duration of urine incontinence, duration of labor, place of delivery, duration of catheterization, delivery outcome, types of fistula, and size of fistula hole of obstetric fistula women. However, there were no significant differences in survival experience among groups of mode of delivery and surgical approach for obstetric fistula patients.

Moreover, the log-rank test shows that there is a significant difference between rural and urban patients in recovery time. A woman who had a stillbirth required more time to recover than a woman who had a live birth. Also, the recovery time of patients who had less than 14 days of catheterization was shorter than that of patients who had more than 14 days of catheterization (Tables 3 and 4).

#### 3.3.3. Comparison of Log-Logistic Accelerated Failure Time Model and Log-Logistic Inverse Gaussian Shared Frailty Model

From table 7 of the supplementary file, we can observe that the AIC values for both the log-logistic accelerated failure time and log-logistic inverse Gaussian shared frailty models are almost similar but not equal. In this study, in order to compare the efficiency of the models, the AIC was used. The mentioned table indicates that the log-logistic inverse Gaussian shared frailty model has a minimum AIC (229.677) than the log-logistic accelerated failure time model (AIC = 236.647). This indicates that the log-logistic inverse Gaussian shared frailty model is the most efficient model to describe time to recovery from obstetric fistula. Moreover, checking the adequacy of parametric baselines using graphical methods, the respective plots are given in figure 22 (supplementary file) and the plot for the log-logistic baseline distribution makes a straight line better than the Weibull and log-normal baseline distributions. This evidence also strengthens the decision made by the AIC. Also, the goodness of fit for the model is illustrated using a Cox-Snell residual test in the supplementary file (figure 23).

### 3.4. Log-Logistic Inverse Gaussian Shared Frailty Model Results

Log-logistic inverse Gaussian shared frailty model results show patients whose height was greater than 150 cm; the rate of recovery was increased by 38% (AHR = 0.69, 95% CI = 0.517–0.860) compared with short patients (less than 150 cm). The rate of recovery was decreased by 22% (AHR = 1.22, 95% CI = 0.23–0.78 (1.110, 1.332)) when patients had a large length of fistula compared to a small length of fistula. Also, the rate of recovery was decreased by 28% (AHR = 1.28, 95% CI = 1.175-1.388) for patients who had an extensive width of fistula compared to those with a small width of fistula. Furthermore, the rate of recovery was increased by 80% (AHR = 0.50, 95% CI = 0.33–0.75) for patients who were treated with physiotherapy as compared to nonuse of physiotherapy (Table 5). While obstetric patients with primary education and medium-sized fistulas are not associated with obstetric fistula recovery time, however, after checked by the global test model, both variables are significantly associated with the recovery time of obstetric fistula (Supplementary file table 10).

## 4. Discussion

The main goal of the study was to assess the determinants of time to recovery from obstetric fistula in patients at Mekelle Hamlin Fistula Center. Covariates such as height, age at delivery, educational status, residence, physiotherapy, place of delivery, and type and size of fistula were the significant predictor variables for time to recovery from obstetric fistula. The clustering effect was significant (*p* value = 0.001) in the log-logistic inverse Gaussian shared frailty model. This showed that there was heterogeneity between the zones in the timing of recovery from obstetric fistula patients.

The percentage of patients who had recovered was 89.33%. This outcome is consistent with research done in Addis Ababa and Gondar [15]. This result, however, was higher than that of a study carried out at the Yirgalem Hamlin Fistula Hospital [12]. The difference might be due to study year and design difference. The prior study only had data from one year, whereas the current study has data from five years. The average recovery time from obstetric fistula is nearly the same as studies conducted at Gondar, Yirgalem, and Addis Ababa Hamlin Fistula Hospital Centers [12, 13, 15].

Literate patients recovered faster than patients who were illiterate. Correspondingly, the finding is consistent for the study conducted in Gondar [15]. Likewise, women's educational status has a negative effect on the incidence of obstetric fistula. For instance, a woman having primary education was 89.3% less likely to have obstetric fistula than women who had no education [19]. Moreover, parallel to Jimma University study, this study finding demonstrated that patients with obstetric fistula from rural residents recovered more slowly than those from urban areas. This may be a result of rural patients' lack of knowledge on the availability of obstetric fistula treatment, which may have caused them to delay seeking treatment. This may be supported by late obstetric fistula treatment which is an indicator for delayed recovery [16].

In the present study, obstetric fistula patients who had a height greater than 150 cm had a shorter recovery time compared to patients who had a height of less than 150 cm. This finding is supported by the study conducted at the Yirgalem Hamlin Fistula Center [12]. Additionally, there was a significant difference in recovery time among groups for BMI of patients. A similar finding was revealed from fertility sparing treatments for endometrial cancer and pelvic exenteration surgery patients [20, 21]. The length of recovery time increased along with catheterization time and fistula width. A similar conclusion was drawn from research carried out in Gondar [15], Addis Ababa, and developing countries [14, 22, 23]. Moreover, similar to the results of the present study, a study from Jimma University revealed that RVF patients recover more quickly than VVF patients [24].

Women who received physiotherapy treatment had 0.801 times more chances of recovering from obstetric fistula than those who did not. The possible reason might be that massaging enhances blood flow throughout the body. This is a new finding, and it needs further research to be adopted as a part of the treatment. Finally, this study demonstrated that patients who gave birth at home need more time to recuperate than those who gave birth in a medical facility. This result is consistent with research from Yirgalem and Southeast India [12].

## 5. Conclusion

Three hundred twenty-eight obstetric fistula patients were included in the study. 89.33% were physically cured, and 10.67% were censored. The overall median survival time to recovery from obstetric fistula patients at Mekelle Hamlin Fistula Center is 33 days.

Height, age at delivery, educational level, place of residence, physiotherapy treatment, place of delivery, types of fistula, and size of the fistula hole were statistically significant predictors for the rate of recovery from fistula patients in the log-logistic inverse Gaussian shared frailty model. Therefore, patients with obstetric fistula who lived in rural areas, gave birth at home, and had a large and extensive size of the fistula hole took longer to recover, whereas patients who were taller than 150 cm received physiotherapy treatment, were older than eighteen at delivery, had completed any formal education, and had RVF types of fistula recovered more quickly.

We recommend medical practitioners to encourage institutional delivery and physiotherapy, reduce catheterization time, and treat urinary incontinence. We also recommend the local health bureau to support female education and pregnancy after the age of 18. The survival probability of patients with obstetric fistulas is better predicted by the log-logistic inverse Gaussian shared frailty model. Therefore, it would be good for future researchers to take this model into account.

## 6. Limitations of the Study

The limitation of the study is the exclusion of some of the variables supposed to influence time to recovery from obstetric fistula in patients, such as age at first marriage, female genital mutilation, and antenatal care visits. Because they are not listed on medical cards, these variables are not included in this study. Also, the study used secondary data from a single center.

## Figures and Tables

**Table 1:** Sociodemographic characteristics of obstetric fistula patients at Mekelle Hamlin Fistula Center, January 2015 to January 2020.

Variables	Category	Event (%)	Censored (%)	Total	Percentage (%)
Age	<18	38 (82.61)	8 (17.39)	46	14.02
	18-30	108 (85.04)	19 (14.96)	127	38.72
	≥30	147 (94.84)	8 (5.16)	155	47.26

Residence	Urban	66 (100)	0	66	20.12
	Rural	227 (86.64)	35 (13.36)	262	79.89

Educational status	No education	132 (83.02)	27 (16.98)	159	48.47
	Primary education	127 (95.49)	6 (4.51)	133	40.55
	Secondary and above	34 (94.44)	2 (5.56)	36	10.98

Height	≤150 cm (short)	54 (80.6)	13 (19.4)	67	20.43
	>150 cm (tall)	239 (91.57)	22 (8.43)	261	79.57

Marital status	Married	196 (91.59)	18 (8.41)	214	65.24
	Single	30 (85.71)	5 (14.29)	35	10.67
	Divorced	53 (81.54)	12 (18.46)	65	19.82
	Other	14 (100)	0	14	4.27

Weight	≤50 kg	55 (83.33)	11 (16.67)	66	20.12
	>50 kg	238 (90.84)	24 (9.16)	262	79.88

Body mass index	Underweight	45 (83.33)	9 (16.67)	54	16.46
	Normal weight	229 (91.6)	21 (8.40)	54	16.46
	Overweight	19 (79.17)	5 (20.83)	24	7.32

Economic dependency	Dependent	65 (82.28)	14 (17.72)	79	24.09
	Independent	228 (91.57)	21 (8.43)	249	75.91

**Table 2:** Obstetric and fistula characteristics of obstetric fistula patients at Mekelle Hamlin Fistula Center, January 2015–January 2020.

Variables	Category	Event (%)	Censored (%)	Total	Percentage (%)
Antibiotic use	No	58 (80.56)	14 (19.44)	72	21.95
	Yes	235 (91.8)	21 (8.20)	256	78.05

Physiotherapy use	No	263 (88.55)	34 (11.45)	297	90.55
	Yes	30 (96.77)	1 (3.23)	31	9.45

Parity	Primiparous	49 (81.67)	11 (18.33)	60	18.29
	Multiparous	244 (91.04)	24 (8.96)	268	81.7

Delivery outcome	Stillbirth	56 (81.16)	13 (18.84)	69	21.04
	Alive birth	237 (91.51)	22 (8.49)	259	78.96

Duration of labor	<1 day	193 (92.34)	16 (7.66)	209	63.72
	1-2 days	56 (86.15)	9 (13.85)	65	19.82
	>2 days	44 (81.48)	10 (8.52)	54	16.46

Place of delivery	Health center	198 (91.24)	19 (8.76)	217	66.16
	Home	95 (85.59)	16 (14.41)	111	33.84

Surgery approach	Vaginal	233 (90.31)	25 (9.69)	258	78.66
	Abdominal	60 (85.71)	10 (14.29)	70	21.34

Incontinence	≤3 months	234 (92.13)	20 (7.87)	254	77.44
	>3 months	59 (79.73)	15 (20.27)	74	22.56

Duration of catheterization	≤14 days	242 (91.67)	22 (8.33)	264	80.49
	>14 days	51 (79.69)	13 (20.31)	64	19.51

Mode of delivery	SVD	221 (91.32)	21 (8.68)	242	73.78
	Cesarean delivery	72 (83.72)	14 (16.28)	86	26.22

Types of fistula	VVF	233 (86.94)	35 (13.06)	268	81.72
	RVF	60 (100)	0	60	18.29

Size of fistula hole	Small	68 (98.55)	1 (1.45)	69	21.04
	Medium	46 (97.87)	1 (2.13)	47	14.33
	Large	69 (93.24)	5 (6.76)	74	22.56
	Extensive	110 (79.71)	28 (20.29)	138	42.07

**Table 3:** Comparisons of time to recovery for obstetric fistula patients among sociodemographic characteristics using log-rank test at Mekelle Hamlin Fistula Center, January 2015–January 2020.

Variables	Median recovery time	Log-rank test		
		Chi-square	Df	*p* value
Height				
<150 cm >150 cm	80 28	186.04	1	0.000
Weight				
≤50 kg >50 kg	76 28	143.06	1	0.000
Age at delivery				
<18 18-30 > 30	90 40 21	337.74	2	0.000
Body mass index				
Underweight Normal weight Overweight	84 47 29	206.27	2	0.000
Residence				
Urban Rural	13 37	178.49	1	0.000
Marital status				
Married Single Divorced Other	28 40 80 39	179.8	3	0.000
Educational status				
No education Primary education Secondary and above	47 23 24	187.94	2	0.000
Economic dependence				
Dependence Independence	71 27	201.46	1	0.000

Df: degree of freedom.

**Table 4:** Comparisons of time to recovery for obstetric fistula patients among obstetric and fistula variables using log-rank test at Mekelle Hamlin Fistula Center, January 2015–January 2020.

Variables	Median recovery time	Log-rank test		
		Chi-square	Df	*p* value
Antibiotic use				
No Yes	76 27	178	1	0.000
Physiotherapy use				
No Yes	34 14	16.94	1	0.000
Parity				
Primiparous Multiparous	84 28	148.07	1	0.000
Delivery outcome				
Stillbirth Alive birth	76 28	187.35	1	0.000
Duration of labor				
<1 day 1-2 days >2 days	24 45 86	240.24	1	0.000
Place of delivery				
Health institution Home	24 56	179.8	3	0.000
Mode of delivery				
SVD CD	32 33	0.31	1	0.578
Surgery approach				
Vaginal Abdominal	32 35	0.40	1	0.528
Duration of incontinence				
≤ 3 months > 3 months	27 76	200.31	1	0.000
Duration of catheterization				
≤14 days >14 days	28 82	179.85	1	0.000
Fistula type				
VVF RVF	37 10	138.33	1	0.000
Fistula size				
Small Medium Large Extensive	18 25 29 42	117.16	3	0.000

**Table 5:** Log-logistic inverse Gaussian shared frailty model analysis of obstetric fistula patients at Mekelle Hamlin Fistula Center, January 2015 to January 2020.

Variables	Estimate ($\hat{\beta }$)	EXP ($\hat{\beta }$)	95% CI for exp ($\hat{\beta }$)	SE ($\hat{\beta }$)	*p* value
Height					
<150 cm	1	1	1	1	
>150 cm	-0.3730	0.688	0.517, 0.860	0.0875	0.047^∗^
Age at delivery					
<18	1	1	1	1	
18-30	-0.35650	10.700	0.457, 0.943	0.1238	0.004^∗^
> 30	-0.65023	0.522	0.259, 0.784	0.1338	0.000^∗^
Residence					
Urban	1	1	1	1	
Rural	0.3057	1.357	1.236, 1.479	0.0621	0.000^∗^
Educational status					
No education	1	1	1	1	
Primary education	-0.08094	0.922	0.830, 1.014	0.0469	0.085
Secondary and above	-0.18593	0.830	0.693, 0.967	0.0699	0.008^∗^
Physiotherapy use					
No	1	1	1	1	
Yes	-0.22146	0.801	0.662, 0.940	0.0708	0.002^∗^
Place of delivery					
Health institution	1	1	1	1	
Home	0.15264	1.165	1.034, 1.296	0.0668	0.022^∗^
Fistula type					
VVF	1	1	1	1	
RVF	-0.51585	0.597	0.433, 0.761	0.0838	0.000^∗^
Fistula size					
Small	1	1	1	1	
Medium	-0.03473	0.966	0.836, 1.096	0.0664	0.601
Large	0.19990	1.221	1.110, 1.332	0.0567	0.000^∗^
Extensive	0.24832	1.282	1.175, 1.388	0.0544	0.000^∗^

*θ* = 0.0762, *τ* = 0.0367, *λ* = 0.29, *ρ* = 2.5, and *AIC* = 229.677. Likelihood ratio test of *θ* = 0, chi-*square* = 8.97, and *p* value = 0.001^∗^. $\text{Exp}\left(\hat{\beta }\right)$ indicates acceleration factor. ^∗^Significant at 5% level. 95% CI for $\exp\;\left(\hat{\beta }\right)$: confidence interval for $\exp\left(\hat{\beta }\right)$; SE ($\hat{\beta }$): standard error for $\hat{\beta }$.

## Data Availability

Data is available on request.

## Abbreviations

AIC: Akaike information criterion
AMREF: African Medical Research Foundation
AOR: Adjusted odds ratio
BMI: Body mass index
CI: Confidence interval
COR: Crude odds ratio
EDHS: Ethiopia Demographic and Health Survey
KM: Kaplan-Meier
Pdf: Probability density function
PH: Proportional hazard
PO: Proportional odds
PVF: Power variance function
q-q: Quantile-quantile
RVF: Recto-vaginal fistula
SSA: Sub-Saharan Africa
VVF: Vesico-vaginal fistula.

## Supplementary Materials

Supplementary Materials The supplementary file consists separate graphs of K-M survivor functions for different categorical variables. And the tables which revealed the mean, median, minimum, and maximum recovery time from obstetric fistula are also available. Additionally, parametric model fitness adequacy checking graphs, tables, quantile-quantile plots, and a plot of the Cox-Snell are presented in detail to further illustrate the model fitness using parametric model fitness. Lastly, global test analysis results for educational status and fistula size are presented in the form of table and texts.
