# Supplementary material for: Survival Modeling on the Determinants of Time to Recovery from Obstetric Fistula: The Case of Mekelle Hamlin Fistula Center, Ethiopia
Source: Int J Reprod Med. 2022 Nov 14;2022:8313575. doi: 10.1155/2022/8313575 (PMC9678468; doi:10.1155/2022/8313575)
Supplement: Supplementary Materials — The supplementary file consists separate graphs of K-M survivor functions for different categorical variables. And the tables which revealed the mean, median, minimum, and maximum recovery time from obstetric fistula are also available. Additionally, parametric model fitness adequacy checking graphs, tables, quantile-quantile plots, and a plot of the Cox-Snell are presented in detail to further illustrate the model fitness using parametric model fitness. Lastly, global test analysis results for educational status and fistula size are presented in the form of table and texts. [file 8313575.f1.docx]

## Supplementary material

### Survival of time to recovery from obstetric fistula for different group of patients

0.00

In general, the survivorship pattern of one is lying above another means the group defined by the upper curve has a better survival than the group defined by the lower curve. For instance, Figure 2, shows that the survival rate of time to recovery from obstetric fistula of urban patients is lower than rural obstetric fistula patients.

### Survival of time –to- recovery by place of residence


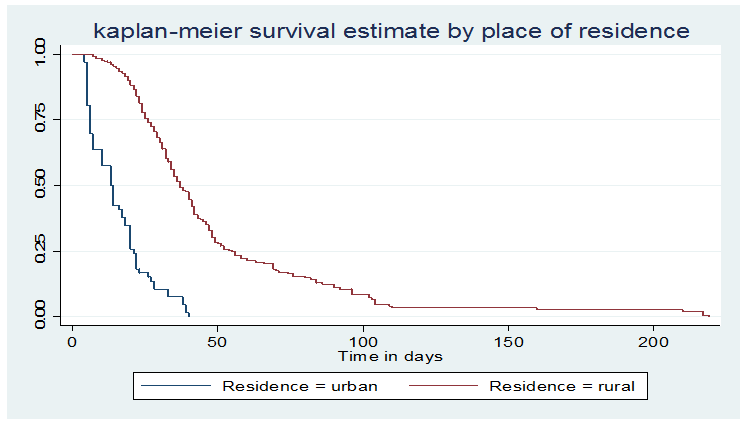
 Figure 2*:* Survival curves of obstetric fistula patients by place of residence

Figure 3*:* Survival curves of obstetric fistula patients by place of delivery

Figure 4*:* Survival curves of obstetric fistula patients by age of delivery

Figure 5*:* Survival curves of obstetric fistula patients by Antibiotic use

Figure 6*:* Survival curves of obstetric fistula patients by fistula size

Figure 7*:* Survival curves of obstetric fistula patients by height

Figure 8***:*** Survival curves of obstetric fistula patients by weight

Figure 9*:* Survival curves of obstetric fistula patients by body mass index

Figure 10*:* Survival curves of obstetric fistula patients by marital status

Figure 11: Survival curves of obstetric fistula patients by educational status

Figure 12*:* Survival curves of obstetric fistula patients by economic dependence

Figure 13*:* Survival curves of obstetric fistula patients by delivery outcome

Figure 14*:* Survival curves of obstetric fistula patients by physiotherapy use

Figure 15*:* Survival curves of obstetric fistula patients by parity

Figure16*:* Survival curves of obstetric fistula patients by duration of labor

Figure 17*:* Survival curves of obstetric fistula patients by mode of delivery

Figure 18*:* Survival curves of obstetric fistula patients by surgery approach

0.00

0.25

0.50

0.75

1.00

0

50

100

150

200

Time in days

Durine = lessthan 3monthes

Durine = more than3monthesl

kaplan-meier survival estimate by duration of urination

Figure 19*:* Survival curves of obstetric fistula patients by duration of urination

Figure 20*:* Survival curves of obstetric fistula patients by duration of catheter

Figure 21*:* Survival curves of obstetric fistula patients by fistula type

Checking adequacy of parametric baselines using graphical methods and AIC result

After the model has been fitted, it is desirable to determine whether a fitted parametric model adequately describes the data or not. Accordingly, their respective plots are given in figure 4.5 below and the plot for the log-logistic baseline distribution make straight line better than Weibull and log-normal baseline distribution. This evidence also strengthens the decision made by AIC value that log-logistic baseline distribution is appropriate for the given dataset.

Figure 22*:* Graph of selected baseline distributions for time to recovery from obstetric fistula.

Table 3: Summary of quantitative dependent variable

| **Variable** | Minimum | Maximum | Mean | Median | Std. deviation |
| --- | --- | --- | --- | --- | --- |
| TTR | 4 | 219 | 42 | 33 | 30.57 |

Std. Deviation: standard deviation and TTR: Time to Recovery measured in day

Table 7: comparison of accelerated failure time models using AIC criteria for obstetric fistula patients

| **Baseline Distribution** | **DF** | **AIC** |
| --- | --- | --- |
| Weibull | 28 | 264.247 |
| Log-normal | 28 | 243.877 |
| Log-logistic | 28 | 236.647 |

Table 8: Comparison of Gamma shared frailty model and inverse Gaussian shared frailty model with different baseline distribution.

| **Baseline Distribution** | **Frailty Distribution** | **AIC Value** |
| --- | --- | --- |
| Weibull | Gamma/inverse gauss | 248.901 /248.779 |
| Log-logistic | Gamma/ inverse gauss | 229.702 /229.677 |
| Log-normal | Gamma/inverse gauss | 234.816 /234.777 |

Table 9: Comparison of log-logistic accelerated failure time model and log-logistic inverse Gaussian shared frailty model

| **Models’ name** | **DF** | **AIC** |
| --- | --- | --- |
| Log-logistic accelerated failure time model | 28 | 236.647 |
| Log-logistic inverse Gaussian shared frailty model | 29 | 229.677 |

AIC= Akaike’s Information Criteria and DF=Degree of Freedom

### Cox-Snell residual plots

A plot of the Cox-Snell residuals against the cumulative hazard is presented in (Figure 24). Cox-Snell residuals are used to assess the overall goodness of fit of the model. If the model fit’s the obstetric fistula data, the plot of cumulative hazard function of residuals against Cox-Snell residuals should be approximately a straight line with slope one. From these plots, the log logistic Cox-Snell residual plot is fairly close to the 45 degree straight line through the origin. This suggests that the model fit to the data is satisfactory. The plot makes straight lines through the origin for log-logistic baseline distribution suggesting that it is appropriate for time-to-recovery from obstetric fistula data.


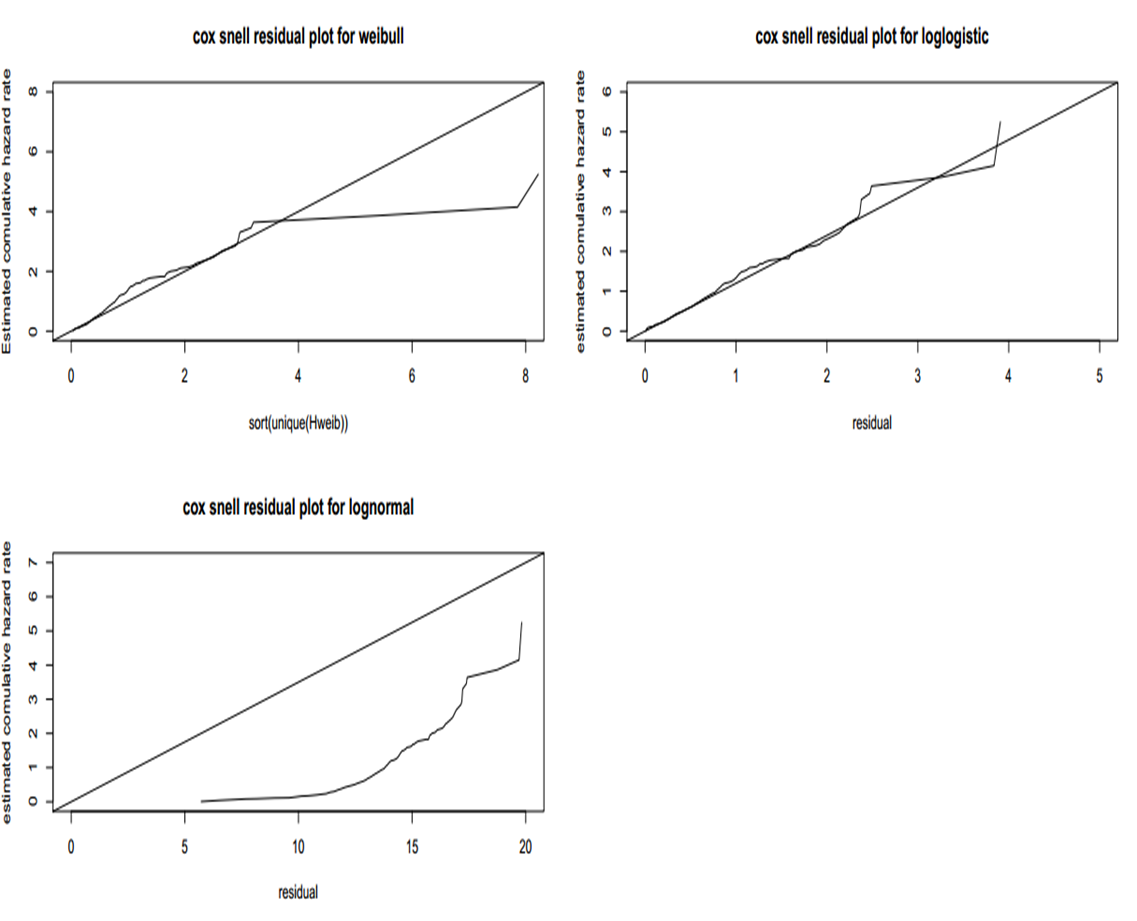


Figure 23: The Cox-Snell residual plots for log-logistic,Weibull and lognormal distributions.

### Quantile-Quantile plot

A quantile-quantile plot is made to check if the accelerated failure time model provided an adequate fit to the data using by two different groups of population. We shall graphically check the adequacy of the accelerated failure time model by comparing the significantly different groups of obstetric fistula patients by place of delivery, education status of patients, place of residence, and size of fistula hole. The figures appear to be approximately linear for all covariates by place of delivery, education status of patients, place of residence for patients, and size of fistula hole of obstetric fistula patients; as shown in Figure below. Therefore, the accelerated failure time appears to be appropriate to describe time-to-recovery from obstetric fistula patients.

Figure 24: q-q plot to check the adequacy of the accelerated failure time model

Global test for some explanatory variables

We have seen that primary level of education and medium size of fistula hole (p-value= 0.747, p-value=0.085) in log-logistic inverse Gaussian shared frailty model (from Table 6) were insignificant levels of predictors. Now, we should check the significances of the predictors (marital status, educational status and fistula size) by using the following global test. That is, if p-value is less than 0.05 we decide that these predictors are significant. From table below we have seen that the p-values (0.000*) of the three predictors (, educational status and fistula size) were less than 0.05 level of significance. Therefore, we can conclude that educational status and fistula size in the log-logistic inverse Gaussian shared frailty model were significant predictors.

Table 10: Global test for marital status and fistula size

| Name predictors | DF | Model’s name | Chi-square | p-value |
| --- | --- | --- | --- | --- |
| Educational status | 2 | Log-logistic IGSF model | 7.35 | 0.0253* |
| Fistula size | 3 | Log-logistic IGSF model | 37.58 | 0.000* |

Where DF=Degree of Freedom, AFT=Accelerated Failure Time and IGSF=Inverse Gaussian Shared Frailty.
